# A Study of Arsenious Acid
*Read before the Philadelphia County Dental Society.


**Published:** 1889-07

**Authors:** L. Ashley Faught

**Affiliations:** Philadelphia, Pa.


ARTICLE VI.

### A STUDY OF ARSENIOUS ACID.*

BY L. ASHLEY FAUGHT, D. D. S., PHILADELPHIA, PA.

Dentistry involving largely a routine mechanical service, its practitioners too often neglect to be thoroughly conversant with the drugs they use. What acquaintance is made with materia medica should be at least sufficiently extended to insure not only safety in use, but ability to cope with the accidental condition of misuse. Not a few of the medicaments employed by dentists are of a poisonous nature, and that to a degree requiring most accurate care in their exhibition, and most prompt and efficient antidotal treatment in cases of injurious action. Perhaps none in this respect demands more careful study than arsenious acid. I invite your attention for a few moments this evening to a definite consideration of the aspects of it, which have heretofore been omitted, or but intimated in a very general manner.

"According to the preparation of the arsenic, and the size of the dose, and the manner of its employment, it is either tonic, antiperiodic, pulmonic, detergent, escharotic, or a *vital* irritant (poison)."† It is ordinarily used in dentistry for pulp devitalization, and for such purpose has been variously combined in mechanical mixture with other materials. The best formula is the old original one.

| | | |
|---|---|---|
| R | Acid arsen. . . . . . . . . . | gr. v |
| | Morph. acet. . . . . . . . . | gr. x |
| | Creosotum vel. ac. carbol. . . | gr. x |
| | M | |

I have never found the other formulæ, subsequently suggested, to possess any additional qualities of which I

* Read before the Philadelphia County Dental Society.

† Dental Cosmos. Vol. XIX, p. 227. Flagg.

did not have the benefit in the one mentioned; while I have, in many, suspicioned the engraftment of undesirable features.

It is not my intention to give my fellow-practitioners instructions in the proper use of arsenious acid; for if any are in need of lessons, the pages of our text-books are adequate, and to them they are respectfully referred. The province of this paper is to record a few facts gathered from experience, so that any seeking information may not in the future search the professional literature for them in vain, as I have done.

In the first place, I desire to suggest that accuracy be used in referring to the drug, naming it as arsenious acid, and not as arsenic, which is so frequently done, for it is an oxide of arsenic, As 2O3; also, that as arsenious acid is poisonous in the highest degree, it is well for everyone using it about the mouth, to acquaint himself with the bulk of the *usual* safe dose, when internally administered. This amount is one-twentieth of a grain, safety existing between the one-sixtieth and one-twelfth of a grain, according to the susceptibility or idiosyncrasy of the patient.

Knowing this quantity definitely, it is positive that systemic trouble cannot supervene, either through local absorption or in the event of the dressing becoming dislodged and swallowed; for "the quantity of arsenious acid to be employed for devitalization will depend upon the structure and class of the tooth, varying from the one-twentyfifth to the one-twentieth of a grain."*

Reference is here made to these points at the risk of being thought over-precise, because it is my belief, sustained by observation, that from our constant contact with the drug there is too often a tendency to carelessness in its use.

So much for the drug itself; I now desire to speak of ways by which arsenious acid is unintentionally intro-

---

* Gorgas' Dental Medicine, p. 130

duced into the mouth, and followed not infrequently by pathological conditions.

The first of these is by uncleanly instrumentation. Care should be taken that the napkins, instruments, vessel containing the medicament, etc., used in making an application, are thoroughly separated from all others, and from contact with the operating tray until cleansed of every trace of the preparation.

I have seen many cases of arsenical ulceration occurring in the mouths of patients in which no arsenious acid had been used; and which were due in all probability to the use of instruments that had previously been employed in connection with arsenious acid in another mouth, and returned to the operating case without thorough cleansing; or to the use of instruments that by virtue of their character were supposed to have no possible connection with arsenious acid, but to which the drug had been transferred from contact with others contaminated, or with the spot on the tray on which they had rested.

The second way is by the use of rubber dam. Some nine months ago I had an experience in this direction. Quite a number of my patients complained, in immediate succession, of sore mouths. Examination revealed the puzzling condition of arsenical ulceration, when I knew that my arsenious acid had not been out of the medicine case for a month.

Recognizing, from previous experience and from the fact that the diseased condition existed only in mouths in which I had been operating with rubber dam, that the cause was the dam, I made application of it to a mouth and produced similar symptoms. The discontinuance of further use of that piece of rubber dam was followed by a cessation of the trouble. I now permanently ceased to use that make of dam, and fixed for the future upon the product of another standard house.

For nine months I have enjoyed immunity, but within the last week the trouble has recurred. From these and like occurrences in past years, the conclusion is drawn that no make of dam can be relied upon as absolutely safe from arsenical taint, and that ulceration may follow its use at any time when circumstances combine to bring together a piece so infected (through some irregularity in its manufacture) and a susceptible mouth. It is, therefore, to be earnestly recommended that each piece be thoroughly washed with soap and water before application.

All that is here written, I am well aware, is opposed by the manufacturers of rubber dam, who make an emphatic denial, and say that preparations of arsenic are never used in its production.

As, however, my clinical evidence is indisputable, they certainly must be in error, or the arsenic is unintentionally introduced by contaminate combination with the chemicals used, such as sulphur, soapstone, etc. The latter may possibly be contaminated by the arsenite of calcium, while sulphur is so frequently contaminated by arsenic, that the greatest possibility exists of such introduction by this means.

The third method of accidental introduction occurs through a combination of conditions. I have met more than one with arsenical ulcerations, due to the escape of the medicament upon the removal of old plugs from teeth, in which the pulps had been devitalized at the time of their insertion. In these cases it is evident that the previous operator failed to remove all traces of arsenious acid before dressing and filling the tooth, and it had remained there inert for many years.

The fourth way is through perforations. Teeth have been tapped to relieve the symptoms of incipient abscess which were due to death of the pulp in but one canal of the tooth, and tapped too far, producing an unrecognized perforation. Arsenious acid has then been applied in what was supposed to be a closed cavity—to destroy the vitality of the remaining portion of the pulp—and has escaped through the perforation.

The last way to which I shall refer in this connection is that with which the dental profession is most familiar—the escape of the drug application by the use of unsuitable coverings to secure it. Writing on the subject of the drug, Dr. Louis Jack says: "Cotton with which wax has been combined, as recommended by Dr. Maynard, cotton partially saturated with sandarach varnish, and gutta percha are each admirable when the circumstances permit."* Prof. James Truman writes, "Cover this with a lead cap, and then fill the cavity with gutta percha."† Notwithstanding the wholesale condemnation which is so frequently made of the use of cotton saturated with sandarach, your essayist has found that his experience agrees with that of Dr. Jack; but he also disagrees with him, with Dr. Truman, and with all others advocating the use of gutta-percha. My experience has been that it is more or less leaky, so that I prefer cement, Dr. Gilbert's temporary stopping, or some similar preparation.

The influence of arsenious acid, when introduced into the mouth so that it comes in contact with the mucous membrane, is as insidious as it is destructive. At the moment of escape there is no evidence of the fact, the local effects becoming manifested in from twenty-four to forty-eight hours. There being no pain, the destruction of tissues is well progressed before the case comes to the operator's notice. The diseased membrane then presents diagnostic points varying with the interval which has elapsed between the escape of the drug and the commencement of treatment. If seen early, the membrane has an appearance somewhat as though it had been scratched with a sharp instrument, the wound being red like a piece of raw beef, while the edges and membrane surrounding show simply deepening of the natural color of the gum— a conjested condition. At a later period the veins in the surrounding tissues may become prominently developed. If they do not share in the congestion to this extent, the trouble will be of short duration. At the expiration of four days, the wounds have the appearance of true ulcers—a deep depressed centre with raised edges—and while not increasing

* American System of Dentistry. vol. II, p. 137.
† American System of Dentistry. Vol. I, p. 902

markedly in area, do increase in depth. The action of arsenious acid seems to destroy, in the first attacked area, by dropping to the bottom of the wound, thus keeping in constant contact with fresh tissue.

Our attention is usually called to arsenical ulcerations by the patient complaining in a similar strain to this: "My gums feel a little tender since the last operation—they hurt a little in brushing the teeth." They do not complain of pain unless the arsenious acid has access to the periosteum. The pain is then severely acute, with all the symptoms of periostitis very much aggravated.

The differential diagnosis between arsenical ulceration and rodent ulcers most commonly found in the mouth is not difficult. In the first place rodent ulcers are generally situated on the soft tissues, inside of the lips, or near the fræmun of the tongue; while arsenical ulcerations have no definite location, and may occur anywhere in the mouth, but are especially prone to be located upon the gums. Then, again, they differ markedly as to appearance. Rodent ulcers are apt to be large and quite painful, while arsenical ulcers are irregular in shape, and like a scratch. Rodent ulcers have a white base, while arsenical ulcers are red, look like raw beef, and are not painful to the touch. With these marked points of difference in appearance, none need err in making a correct diagnosis.

TREATMENT.—Whenever practical, the soft tissues should be curetted,—scarify freely, wash away the blood and shredded membrane with warm water, and then touch the wound with muriate tincture of iron, or cover with magnesia (Husband's). Then cauterize the wound with carbolic acid, or apply tincture of iodine, and if needed stimulate further in a few days by another application of the last used, "remembering that if we produce inflammation of a part, we check its absorptive power."* The attack is usually fully ended in about ten days, and where the treatment here indicated is promptly and thoroughly used, I have never seen the patient much inconvenienced, or the tissue much destroyed;—in other words, no untoward results have followed.—*International Dental Journal.*

* Farquharson's Therapeutics and Materia Medica, p. 152.

